# Mining bone metastasis related key genes of prostate cancer from the STING pathway based on machine learning

**DOI:** 10.3389/fmed.2024.1372495

**Published:** 2024-05-21

**Authors:** Guiqiang Li, Runhan Zhao, Zhou Xie, Xiao Qu, Yingtao Duan, Yafei Zhu, Hao Liang, Dagang Tang, Zefang Li, Weiyang He

**Affiliations:** ^1^Department of Urology, The First Affiliated Hospital of Chongqing Medical University, Chongqing, China; ^2^Department of Urology, Chongqing Traditional Chinese Medicine Hospital, Chongqing, China; ^3^Department of Orthopedics, The First Affiliated Hospital of Chongqing Medical University, Chongqing, China; ^4^Department of Orthopedics, Chongqing Traditional Chinese Medicine Hospital, Chongqing, China; ^5^Department of Orthopedics, Qianjiang Hospital Affiliated with Chongqing University, Chongqing, China

**Keywords:** prostate cancer, bone metastasis, random forest, STING, nomogram

## Abstract

**Background:**

Prostate cancer (PCa) is the second most prevalent malignant tumor in male, and bone metastasis occurs in about 70% of patients with advanced disease. The STING pathway, an innate immune signaling mechanism, has been shown to play a key role in tumorigenesis, metastasis, and cancerous bone pain. Hence, exploring regulatory mechanism of STING in PCa bone metastasis will bring novel opportunities for treating PCa bone metastasis.

**Methods:**

First, key genes were screened from STING-related genes (SRGs) based on random forest algorithm and their predictive performance was evaluated. Subsequently, a comprehensive analysis of key genes was performed to explore their roles in prostate carcinogenesis, metastasis and tumor immunity. Next, cellular experiments were performed to verify the role of RELA in proliferation and migration in PCa cells, meanwhile, based on immunohistochemistry, we verified the difference of RELA expression between PCa primary foci and bone metastasis. Finally, based on the key genes to construct an accurate and reliable nomogram, and mined targeting drugs of key genes.

**Results:**

In this study, three key genes for bone metastasis were mined from SRGs based on the random forest algorithm. Evaluation analysis showed that the key genes had excellent prediction performance, and it also showed that the key genes played a key role in carcinogenesis, metastasis and tumor immunity in PCa by comprehensive analysis. In addition, cellular experiments and immunohistochemistry confirmed that overexpression of RELA significantly inhibited the proliferation and migration of PCa cells, and RELA was significantly low-expression in bone metastasis. Finally, the constructed nomogram showed excellent predictive performance in Receiver Operating Characteristic (ROC, AUC = 0.99) curve, calibration curve, and Decision Curve Analysis (DCA) curve; and the targeted drugs showed good molecular docking effects.

**Conclusion:**

In sum, this study not only provides a new theoretical basis for the mechanism of PCa bone metastasis, but also provides novel therapeutic targets and novel diagnostic tools for advanced PCa treatment.

## 1 Introduction

Prostate cancer (PCa) is the second most prevalent malignant tumor in the male population, with about 20% of male individuals developing the disease at some point. As a highly aggressive tumor, most advanced PCa patients are diagnosed with multiple metastasis throughout the body, mainly in lymph nodes near the prostate, distal lymph nodes, bone, as well as internal organs such as the liver, lungs, and brain (1, 2). Among the distal metastasis of PCa, bone is most common site of colonization, and approximately 70% of advanced PCa patients are diagnosed with bone metastasis (3). Once bone metastasis occurs, the disease is incurable and is significantly associated with mortality (4–6). Tumor growth in the bone can cause pain, hypercalcemia, anemia, fracture, and other adverse events, all of which severely impact the patient’s survival status and quality of life (7, 8). The tumor bone pain usually presents as a persistent dull ache that increases in intensity over time and reaches a level not relieved by opioids (9, 10). Neurological dysfunction, pain, anxiety and depression due to bone metastasis are devastating for patients, severely affecting their quality of life and significantly increasing mortality (7, 9, 11–13). With the continuous iterative updating of treatment regimens, the median survival of PCa patients has been significantly prolonged, leading to an elevated incidence of bone metastasis, making this phenomenon even more clinically relevant (14). However, the current detection of metastasis is minimal, and it is estimated that only about 0.02% of cancer cells entering the blood circulation will produce clinically detectable metastasis (15). Once metastasis occurs, it is responsible for approximately 90% of all deaths (16). Therefore, there is an urgent need to increase our understanding of the cellular and molecular mechanisms involved in PCa bone metastasis to improve the prognosis of patients with bone metastasis.

As studies have progressed in recent years, researchers have found a strong link between tumor immunity and bone metastasis. Due to the strong similarities with inflammation, cancer has long been described as a wound that cannot be healed (17, 18). During routine wound healing, the body terminates the immune response in a timely manner through various immune regulatory mechanisms (19), whereas in tumors, the uncontrolled inflammatory response becomes a powerful driver of tumorigenesis (20–22). The STING pathway is a vital transduction mechanism in innate immunity and viral defense, and it also plays a crucial role in carcinogenesis and development (23). Substantial evidence that STING activators (DMXAA and ADU-S100) can inhibit tumor progression and increase survival in an adaptive immune cell-dependent manner (24–27). Studies have shown an essential link between chromosomal instability and tumor metastasis and that cell membrane dsDNA produced by persistent chromosome segregation errors is sensed by the STING pathway (28, 29). In brain metastasis from breast cancer, tumor cells communicate with adjacent astrocytes by producing cAMP signals that activate the STING signaling pathway to release inflammatory factors, leading to tumor and metastasis progression (30). In addition, in prostate, breast, and lung cancers, STING signaling can also promote or inhibit the onset and progression of bone metastasis by modulating immune cells (31–35). Based on synergistic effects on injury receptors, immune cells, and osteoclasts, the STING pathway is also significant in regulating cancerous bone pain (36). Since the STING pathway plays a critical role in bone metastasis, exploring its regulatory mechanism in PCa bone metastasis will bring novel opportunities for treating PCa bone metastasis.

In this study, we mined three key genes related to PCa bone metastasis from STING-related genes (SRGs) based on the random forest machine learning algorithm, constructed an accurate nomogram, and discovered several targeted drugs for key genes. These findings provide novel ideas to improve treatment strategies for patients with advanced PCa.

## 2 Methods

### 2.1 Cell culture and transfection

The human PCa cell line (DU145 and PC3) was purchased from the America Type Culture Collection (ATCC, United States). Meanwhile, the cells were inoculated in 1640 medium (Saimikebio; China) containing 10% FBS (ExCell Biology, Inc., Shanghai, China) and 1% penicillin–streptomycin (100 IU/mL; Hyclone; Cytiva), and then placed in certain environments (37°C and 5% CO2) to culture. The RELA-overexpression plasmid was purchased from YouBio Biotechnology Co., Ltd. (Hunan, China). Transfection was performed using lipofectamine 3000 (Invitrogen) according to the manufacturer’s instructions.

### 2.2 Cell proliferation assay

Cell proliferation was measured using the CCK-8 assay. Inoculate DU145 and PC3 cells transfected with RELA-overexpression plasmid and transfected with GFP control plasmid in 96-well plates (5,000 cells/well). After a period of incubation, 10ul of CCK-8 solution was added to each well. Optical density (OD) value was evaluated using a microplate reader at 450 nm 2 h later.

### 2.3 Cell migration assay

Cell migration was assessed by transwell assay. 2.5 × 10^4^ DU145 and PC3 cells transfected with RELA-overexpression plasmid and transfected with GFP control plasmid were inoculated into the upper chamber of the transwell. After 24 h of incubation in the incubator, use a moistened cotton swab to carefully wipe off the cells that did not pass through the holes, and then add crystal violet to stain the chamber and take photographs.

Cell migration was also measured using wound healing assay. DU145 and PC3 cells transfected with RELA-overexpression plasmid and transfected with GFP control plasmid were inoculated in 6-well culture plates. After the cell fusion rate reached 90%, the cell layer was scratched using the tip of a sterile lance tip. Next, after washing with PBS, the culture was continued with serum-free 1,640 medium, and the fixed sites were photographed using a light microscope at 0, 12, and 24 h.

### 2.4 Immunohistochemistry

Paraffin sections of PCa primary foci and bone metastasis were obtained from the Department of Pathology with the approval of the Ethics Committee of our Medical Center. First, the paraffin sections were dewaxed in an environmentally friendly dewaxing solution and hydrated in a gradient ethanol series, and heat-mediated antigenic repair was performed on them in a microwave oven using the citric acid antigen repair solution; next, the sections were incubated with an endogenous peroxidase blocking agent (3% H_2_O_2_) for 25 min at room temperature and protected from light; subsequently, circles were drawn with a immunohistochemical pen, and section sealing was operated with 3% BSA; Finally, immunohistochemical staining was performed according to standard procedures.

As for the immunohistochemical staining operation. First, paraffin sections were incubated with RELA primary antibody (Servicebio, GB11997, 1:1,000) overnight at 4°C; next, sections were incubated with secondary antibody (goat anti-rabbit IgG) for 50 min at room temperature; subsequently, sections were stained sequentially using DAB and hematoxylin; finally, the stained sections were dehydrated in ethanol and xylene and sealed with neutral resin. Immunohistochemical images were obtained based on a light microscope (E100, Nikon, Japan).

### 2.5 Data collection and preprocessing

GEO^1^ is a free and publicly available gene expression database containing many diseases. We downloaded the gene expression dataset (GSE32269) containing PCa primary foci and bone metastasis from this database. Based on the annotation files of the corresponding platforms, we matched probes to their gene symbols on the dataset. Those with larger mean values were selected for retention when duplicate probes existed. Subsequently, based on a clustering algorithm, outlier samples were removed before subsequent analysis. Finally, 103 SRGs were obtained from the GeneCards database.^2^

### 2.6 Identification and evaluation of key genes

The SRGs were initially downscaled by a univariate logistic regression algorithm, and the genes that satisfied the *p*-value < 0.05 were considered candidate genes. Next, the candidate genes were assigned MDA and MDG values based on the random forest algorithm. MDA and MDG are two key indicators for assessing the importance of a variable by the random forest algorithm, and the larger of these two indicates the higher importance of the corresponding variable. Subsequently, genes ranked in the top 5 of MDA and MDG were cross-analyzed to filter out key genes. Finally, the predictive performance of the key genes was assessed by the Receiver Operating Characteristic (ROC) curve and confusion matrix.

### 2.7 Biological function exploration of key genes

First, Gene Ontology (GO) analysis, Kyoto Encyclopedia of Genes and Genomes (KEGG) analysis, and Gene set enrichment analysis (GSEA) analysis were performed to initially explore the key genes’ biological functions. Subsequently, correlation and differential expression analyses were performed to explore the internal connections of the key genes and their function in PCa with bone metastasis. Then, based on the intOGen database^3^ and the Depmap database,^4^ we explored the role of key genes in PCa carcinogenesis. Subsequently, through the Gene Set Cancer Analysis database (GSCA),^5^ we explored the regulation of key genes by copy number variation (CNV) and methylation. Finally, regulatory miRNAs for key genes included in miRecords, miRTarBase, and TarBase databases were obtained based on the “multiMiR” R package, and miRNA-mRNA regulatory networks were mapped using the Cytoscape software (V 3.9.1).

### 2.8 Immune analysis of key genes

Here, the single sample gene set enrichment analysis (ssGSEA) algorithm was used to calculate the degree of infiltration for 28 types of immune cells for each sample in the dataset. And by observing the immune microenvironment differences between primary and bone metastatic foci, the correlation between immune cells and metastasis, and the correlation between key genes and immune cells, we aimed to unearth the influence of key genes on PCa bone metastasis at the immune level. Meanwhile, the differential expression of several common immune checkpoints between primary and metastatic foci and the correlation between key genes and immune checkpoints were explored, aiming to explore the value of key genes in immunotherapy.

### 2.9 Molecular functional validation of RELA

Bioinformatic analysis showed that all three key genes play critical roles in prostate cancer bone metastasis, among which RELA is of particular attention. Therefore, in this study, the molecular function of RELA in prostate cancer metastasis was verified based on cell proliferation and migration assays after overexpression of RELA in prostate cancer cells using the RELA plasmid. Finally, the protein expression of RELA was explored by immunohistochemistry between PCa primary foci and bone metastasis foci, aiming to validate the differential expression of this gene between different pathological tissues at the protein level.

### 2.10 Construction of nomogram and prediction of targeted drugs

At the end of this study, we constructed a nomogram based on key genes, aiming to provide a new diagnostic tool for clinicians. Meanwhile, the accuracy and reliability of the nomogram were evaluated and validated by ROC curve, Calibration curve, and Decision Curve Analysis (DCA) curve. In addition, to further validate the predictive performance of the three key genes and the nomogram, we performed 200 times 5-Fold cross-validation. Next, the targeted drugs for the key genes were predicted based on the Enrichr database^6^ (37). Subsequently, molecular docking was performed between targeted drugs and key genes with top 5 combined score using the Ledock software. The 3D structure data of proteins and targeted drugs were obtained from the RSCB-PDB database^7^ and the PubChem database,^8^ respectively. Finally, molecular docking was visualized using the Pymol software.

## 3 Results

### 3.1 Identification and evaluation of key genes

**Figure 1:**
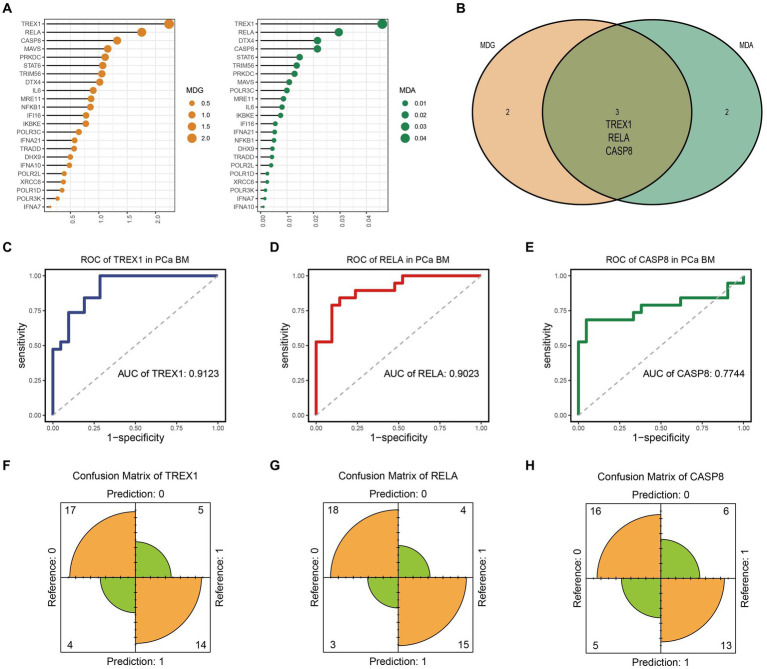
Identification and evaluation of key genes. **(A)** MDA and MDG ordering of candidate genes; **(B)** Identification of key genes; **(C–E)** ROC curve of TREX1, RELA, and CASP8; **(F–H)** Confusion matrix of TREX1, RELA, and CASP8.

Here, we obtained gene expression data of 84 SRGs from GSE32269 dataset. Meanwhile, 40 samples were included by cluster analysis for this analysis (Supplementary Figure 1). Based on the univariate logistic regression algorithm, we performed an initial downscaling of the 84 SRGs and 24 genes with *p*-value < 0.05 were identified as candidate genes (Table 1). Subsequently, the MDA and MDG values of the 24 genes were calculated using the random forest algorithm, and genes with negative MDA or MDG values were excluded (Figure 1A). Then, we performed cross-tabulation analysis on the genes ranked in the top five of MDA and MDG and finally obtained three key genes (TREX1, RELA, and CASP8), which was shown in Figure 1B.

**Table 1:** Univariate logistic regression results of 24 candidate genes.

Gene	OR	CI (5–95%)	*P*-value
XRCC6	0.03	0–0.47	0.01
TRIM56	0.1	0.02–0.44	0
TREX1	0	0–0.15	0
TRADD	0.08	0.01–0.56	0.01
STAT6	0.36	0.15–0.83	0.02
RELA	0	0–0.04	0
PRKDC	8.66	2.02–37.12	0
POLR3K	6.82	1.26–37.01	0.03
POLR3C	13.6	1.7–108.7	0.01
POLR2L	12.78	2.01–81.3	0.01
POLR1D	3.03	1.04–8.83	0.04
NFKB2	3.81	1.06–13.76	0.04
NFKB1	0.01	0–0.23	0
MRE11	11.26	2.35–54.03	0
MAVS	8.79	1.58–48.96	0.01
IL6	0.07	0.01–0.44	0
IKBKE	5.05	1.32–19.24	0.02
IFNA7	3.71	1–13.81	0.05
IFNA21	3.5	1.18–10.4	0.02
IFNA10	5.06	1.59–16.15	0.01
IFI16	3.49	1.32–9.19	0.01
DTX4	9.65	1.97–47.17	0.01
DHX9	2.53	1.25–5.11	0.01
CASP8	4.75	1.26–17.91	0.02

Finally, we evaluated the prediction accuracy of the three key genes based on the ROC and confusion matrix. The ROC curves showed that the AUC values of the three key genes were 0.9123, 0.9023, and 0.7744, respectively, which indicated that the three key genes had a high prediction accuracy (Figures 1C–E). In addition, as shown in Figures 1F–H, the confusion matrix also indicated that the key genes had good prediction performance.

### 3.2 Biological function exploration of key genes

**Figure 2:**
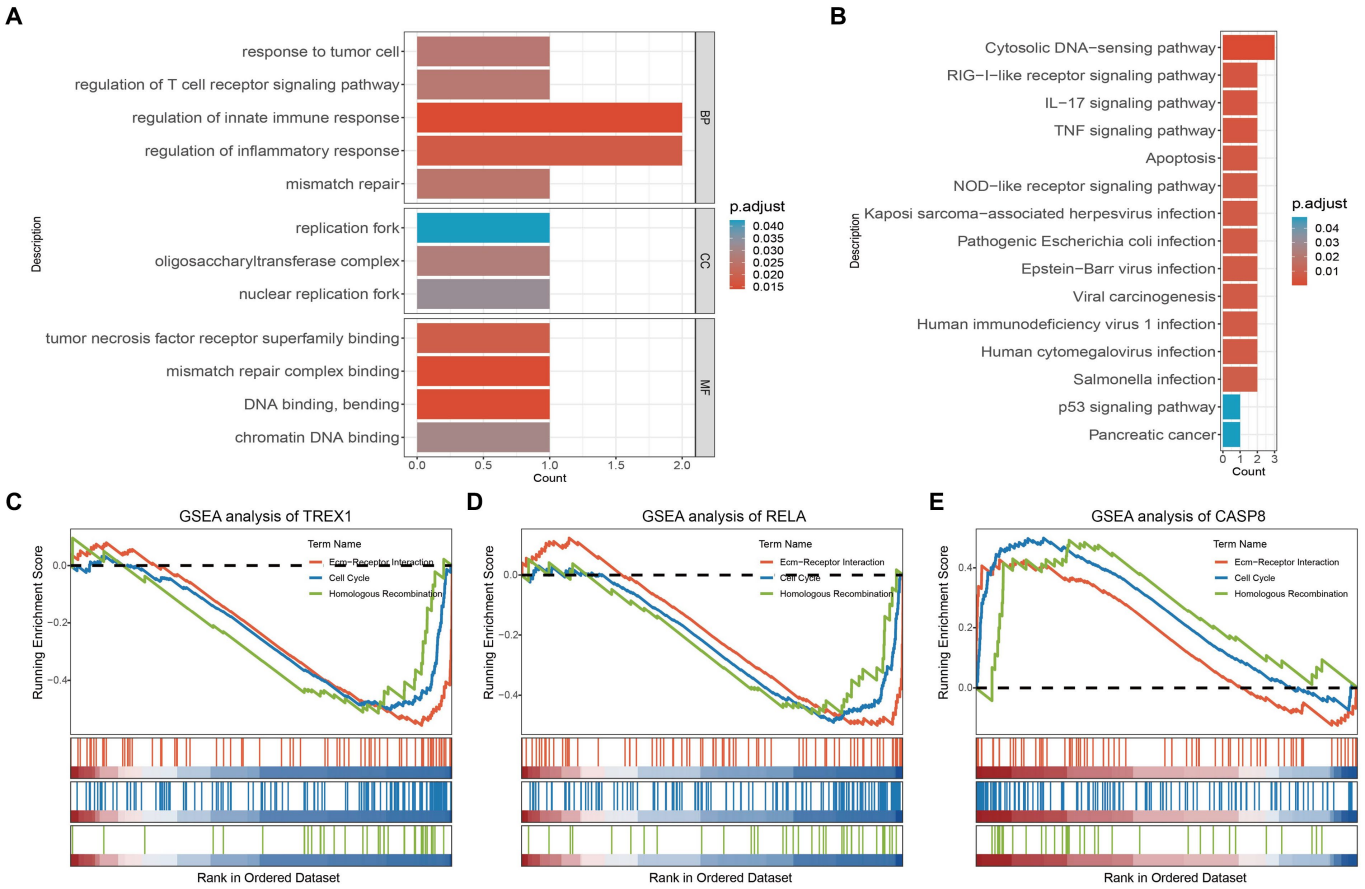
GO, KEGG and GSEA analysis of key genes. **(A)** GO analysis of key genes; **(B)** KEGG analysis of key genes; **(C–E)** GSEA analysis of key genes.

To explore the biological functions of the key genes, we performed GO analysis, KEGG analysis, and GSEA analysis. As shown in Figure 2A, GO analysis showed that the key genes were mainly involved in response to tumor cell, regulation of T cell receptor signaling pathway, regulation of innate immune response, mismatch repair (BP, Biological Process); replication fork, oligosaccharyltransferase complex, nuclear replication fork (CC, Cell Component); tumor necrosis factor receptor superfamily binding, mismatch repair complex binding, DNA binding, bending, chromatin DNA binding (MF, Molecular Function). The results suggest that key genes are involved in the regulation of tumors as well as immune-related mechanisms. Furthermore, KEGG also confirmed this result from another aspect. The KEGG analysis showed that the key genes were mainly enriched in tumor and immune-related signaling pathways, such as the TNF signaling pathway, Viral carcinogenesis, p53 signaling pathway, Pancreatic cancer, RIG−I−like receptor signaling pathway, IL-17 signaling pathway, etc. (Figure 2B). It is well known that Ecm-Receptor Interaction, Cell Cycle, and Homologous Recombination are key signaling pathways in tumorigenesis. And the Ecm-Receptor Interaction also plays a crucial role in PCa bone metastasis (38–40). Subsequent GSEA analysis showed that TREX1 and RELA inhibited the activation of Ecm-Receptor Interaction, Cell Cycle, and Homologous Recombination, while CASP8 was involved in the activation of these three signaling pathways (Figures 2C–E).

**Figure 3:**
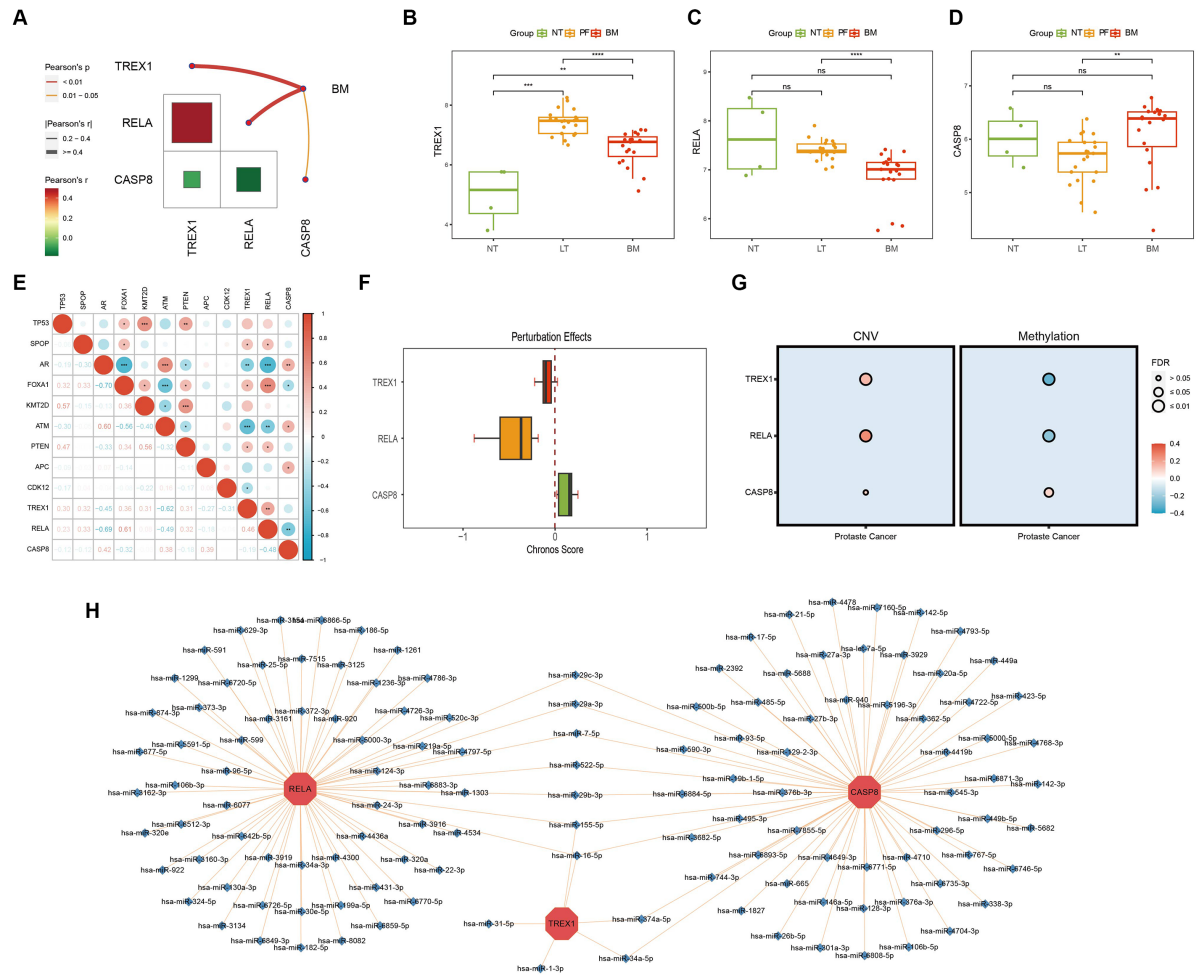
Biological function exploration of key genes. **(A)** Internal correlation analysis of key genes; **(B–D)** Differential expression analysis of key genes (NT, normal tissue; PF, primary foci; BM, bone metastasis); **(E)** Correlation analysis of key with PCa driver genes; **(F)** Chronos Score of key genes; **(G)** Correlation analysis of key genes with CNV/methylation; **(H)** miRNA-mRNA regulatory network of key genes.

Correlation analysis showed a strong internal correlation between the three key genes and was closely associated with bone metastasis (Figure 3A). Differential expression analysis showed that three key genes had significant expression differences in primary foci and bone metastatic foci tissues (Figures 3B–D), demonstrating that the key genes play critical roles in developing PCa bone metastasis. Tumor driver genes play a crucial role in tumorigenesis. Chronos Score is a metric in the Depmap database to assess the degree of impact on cell proliferation after gene knockdown, and a more negative value indicates a greater impact of the gene on cell proliferation (41). As shown in Figures 3E,F, the three key genes were significantly correlated with multiple PCa driver genes; and the Chronos Score of TREX1 and RELA, except CASP8, were negative in prostate tumor cells. The above results demonstrated that the three key genes also play critical roles in PCa development.

The CNV, methylation, and miRNA are critical parts of the gene expression regulatory network, hence we also analyzed the effects of CNV, methylation, and miRNA on key genes. The results showed that in CNV, the three key genes were significantly positively correlated with CNV; while in methylation, TREX1 and RELA were significantly negatively correlated with methylation, except for CASP8, which was positively correlated with methylation (Figure 3G); meanwhile, the miRNA-mRNA regulatory network showed that a total of 128 miRNAs were involved in the regulation of three key genes, and 9 miRNAs were involved in the regulation of several key genes, among which has-miR-155-5P and has-miR-16-5P were noteworthy (Figure 3H).

In conclusion, three key genes play an essential role in the development and metastasis of PCa. And in the process of bone metastasis, TREX1 and RELA are protective factors, while CASP8 is a risk factor.

### 3.3 Immune analysis of key genes

**Figure 4:**
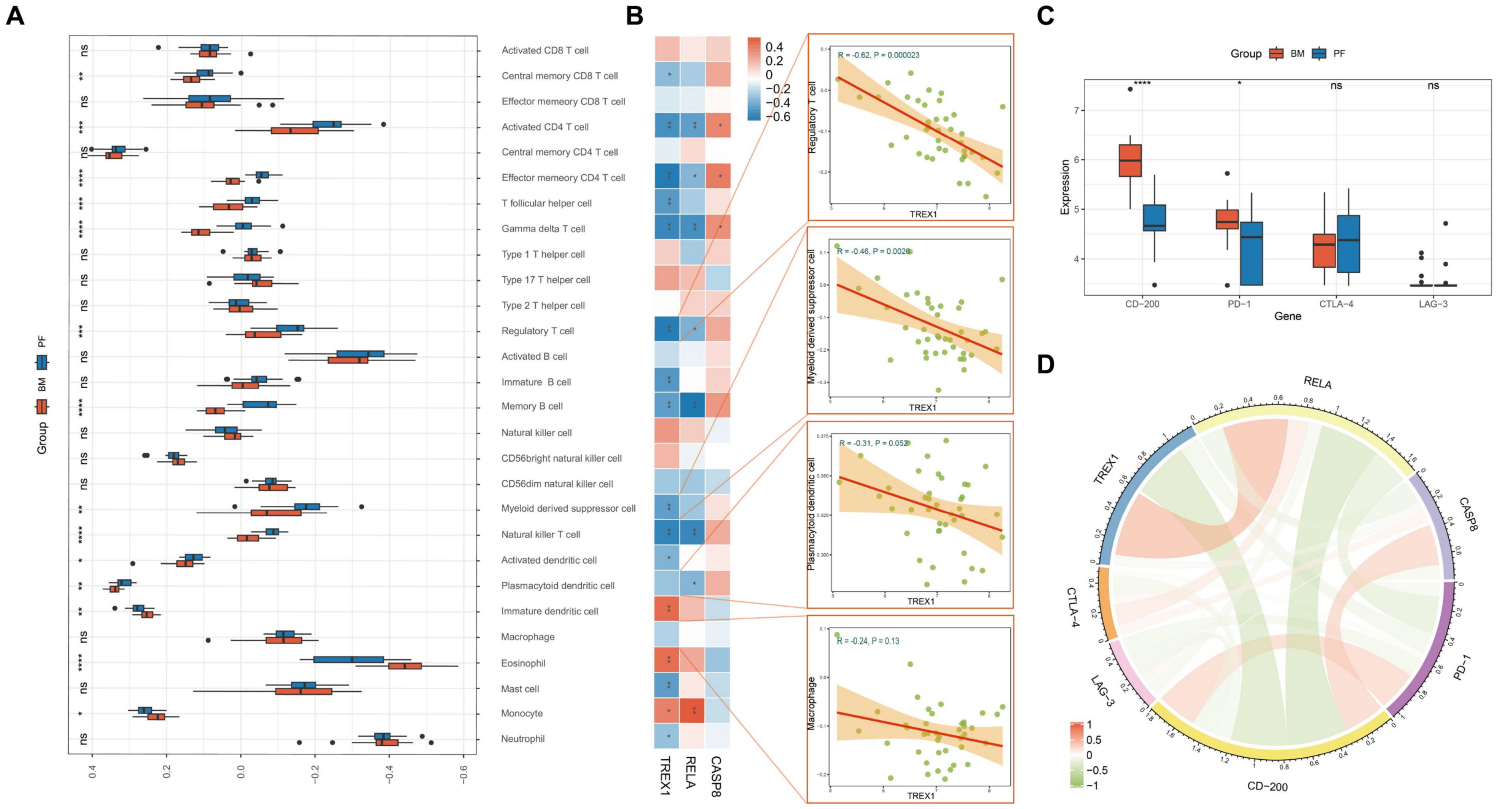
Immune analysis of key genes. **(A)** Differential analysis of the degree of immune cell infiltration in primary foci and bone metastasis (PF, primary foci; BM, bone metastasis); **(B)** Correlation analysis of key genes and immune cells; **(C)** Differential expression analysis of immune checkpoints in primary foci and bone metastasis; **(D)** Correlation analysis of key genes and immune checkpoints.

The above studies revealed that key genes were involved in the immune regulation of tumors, so we explored the key genes in the immune-related mechanisms of PCa bone metastasis. Analysis of immune infiltration differences showed that 14 immune cells had significant infiltration differences between primary and metastatic foci (Figure 4A). Studies have shown that four types of immune cells, Macrophage, Myeloid derived suppressor cell, Regulatory T cell, and Plasmacytoid dendritic cell promote PCa bone metastasis (32, 42–44). Meanwhile, correlation analysis showed that TREX1 was closely associated with a variety of immune cells and was significantly negatively correlated with three types of bone-metastasis-promoting immune cells (Regulatory T cells, Myeloid derived suppressor cells, and Plasmacytoid dendritic cells); RELA was significantly negatively correlated with two types of bone-metastasis-promoting immune cells (Regulatory T cells and Plasmacytoid dendritic cells); whereas CASP8 showed an opposite correlation trends (Figure 4B). Of course, tumor immunity is a very complex biological regulatory network, and a variety of immune cells (CD8 T cells, Natural killer cells, and Monocytes) have been also shown to play important roles in tumor bone metastasis (45–47). The molecular mechanisms between the three key genes and the four bone-metastasis-promoting immune cells as well as other types of immune cells are complex and need to be explored by subsequent in-depth studies.

Immune checkpoints have been a hot research topic in the field of oncology, and in this study, we found that CD-200 and PD-1 were significantly highly expressed in bone metastatic foci (Figure 4C); TREX1 and RELA were negatively correlated with CD-200 and PD-1, while CASP8 was positively correlated with CD-200 and CTLA-4 (Figure 4D). Thus, key genes also play a role in the immunoregulation of bone metastasis in PCa.

### 3.4 Molecular functional validation of RELA

**Figure 5:**
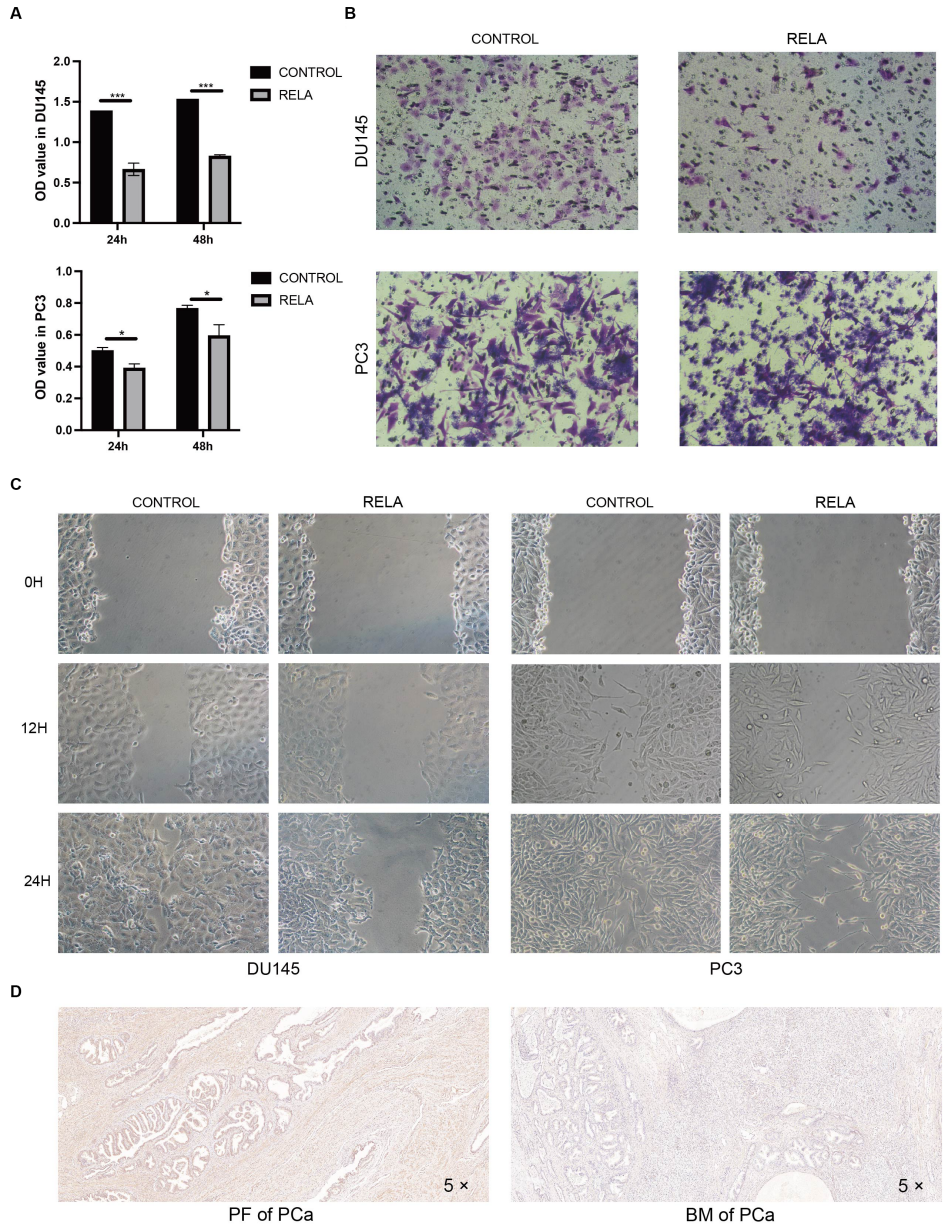
Molecular functional validation of RELA. **(A)** Cell proliferation assessment of DU145 and PC3 cells based on CCK-8 assay (RELA, PCa cells treated with RELA-overexpression plasmid; ^*^p-value < 0.05; ^***^p-value < 0.001); **(B)** Cell migration assessment of DU145 and PC3 cells based on transwell assay; **(C)** Cell migration assessment of DU145 and PC3 cells based on wound healing assay; **(D)** Immunohistochemistry of RELA in the PCa primary foci (PF) and bone metastasis (BM).

The results of several analyses indicate that RELA plays a crucial role in the ontogenesis, progression, and metastasis of PCa. Hence, we validated its molecular function in proliferation and metastasis. Cell proliferation assay showed that the proliferation ability of DU145 and PC3 cells was significantly inhibited after overexpression of RELA (Figure 5A). Meanwhile, cell migration assay showed the same trend. As shown in Figures 5B,C, the migration of DU145 and PC3 cells were also significantly inhibited after overexpression of RELA. In addition, we verified the difference in RELA expression between primary foci and bone metastasis by immunohistochemistry. The results showed that the expression of RELA was significantly lower in bone metastasis compared with primary foci, which was consistent with the results of our data analysis (Figure 5D). Hence, this greatly confirms the critical role of RELA in PCa development and metastasis, and is most likely a novel therapeutic target.

### 3.5 Construction of nomogram and prediction of targeted drugs

**Figure 6:**
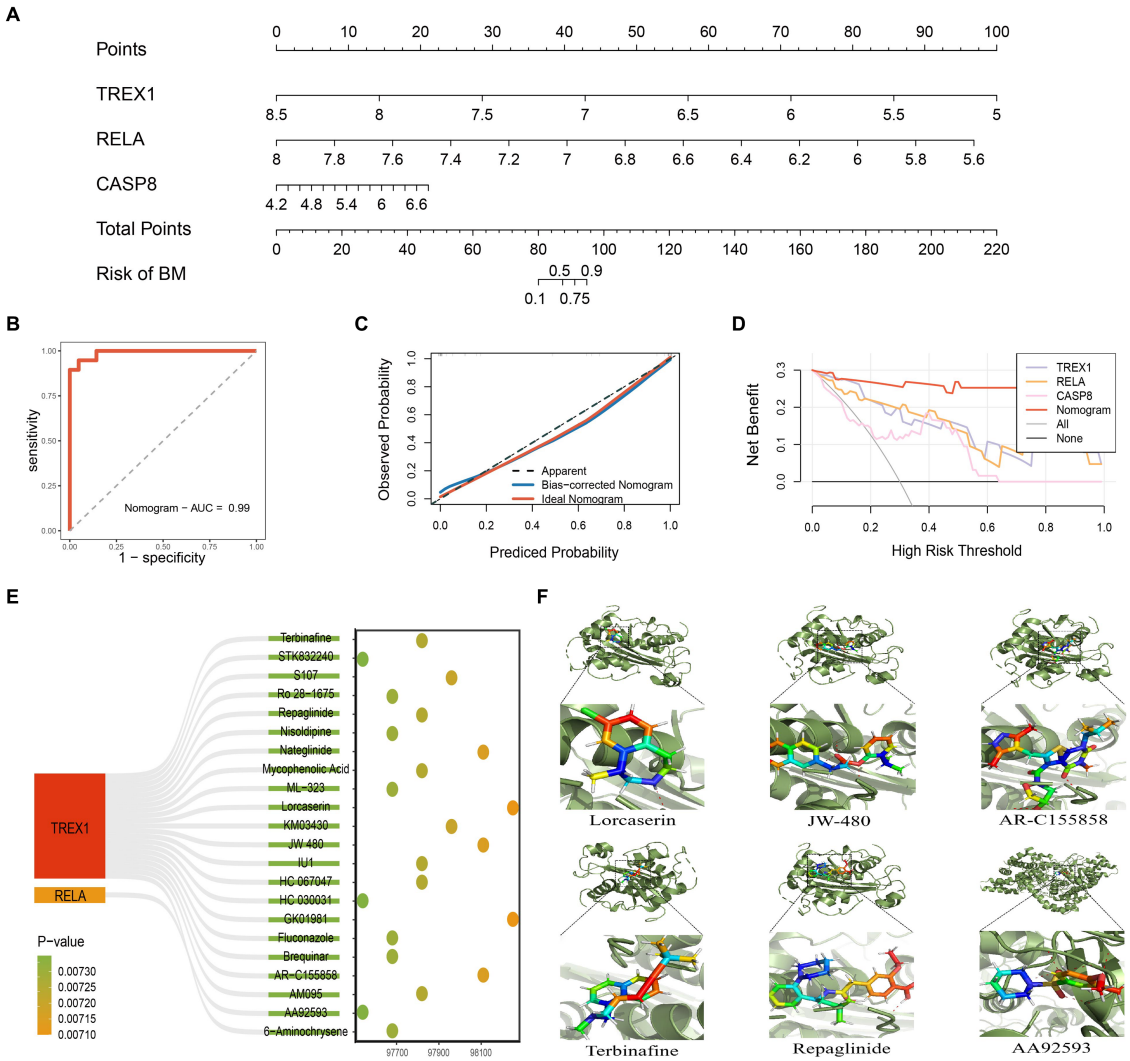
Construction and evaluation of nomogram. **(A)** Nomogram constructed based on key genes; **(B)** ROC curve of the nomogram; **(C)** Calibration curve of the nomogram; **(D)** DCA curve of the nomogram; **(E)** 22 kinds of potential targeted drugs for TREX1 and RELA; **(F)** Molecular docking results for the top 5 TREX1 and RELA-targeted drugs in the combined score.

Based on the three key genes, we constructed a nomogram designed to help clinicians make clinical predictions (Figure 6A). Subsequently, we evaluated the accuracy and reliability of the nomogram. The results showed that the AUC value of the ROC curve was 0.99, and both the Calibration curve and the DCA curve performed well (Figures 6B–D). In addition, a 200 times 5-fold cross-validation analysis suggested high AUC and C-Index values for all three key genes and the nomogram (Supplementary Figure 2). Therefore, the nomogram constructed in this study was accurate and reliable. Next, based on the Enrichr database, we excavated 22 potential TREX1 and RELA targeted overexpression drugs (Figure 6E; Supplementary File 1). Finally, molecular docking was performed using the Ledock software for the targeted drugs with a top 5 ranked TREX1 and RELA combined score. The results showed that the targeting pockets of the 2 key genes were successfully occupied by the drugs (Figure 6F). In conclusion, the nomogram constructed and the new targeted drugs discovered in this study provided a new strategy for the treatment of advanced PCa.

## 4 Discussion

Bone metastasis occur in more than 1.5 million cancer patients worldwide, and the tumors most at risk for this complication are PCa and breast cancer (48). Although there are well-established treatment strategies for early-stage PCa, including surgical resection, chemotherapy, and androgen deprivation (49), most PCa continues to progress and develop bone metastasis. Studies have shown that the incidence of bone metastasis in PCa is about 70%, and the most common site is the vertebrae (50, 51). The development of bone metastasis can be catastrophic for PCa patients, who often suffer from fractures, spinal cord compression, and disability (9, 48), significantly reducing survival quality. Unfortunately, there is no effective treatment for PCa bone metastasis at this stage (52, 53). It is urgent to deeply explore the molecular mechanisms of PCa bone metastasis and tap new therapeutic targets.

It is well known that the immune system plays an indispensable role in maintaining normal bone homeostasis and in various bone-related diseases, mainly through inflammation. Recently, it has been found that pro-inflammatory cytokines in tumors cause homeostatic abnormalities in osteoclasts and osteoblasts, leading to the development of bone metastasis (54, 55). Furthermore, there is also evidence that immune cells can influence the colonization and progression of tumor cells in bone metastatic foci (56). The STING pathway, as an innate immune regulatory mechanism in the human body, has been shown to play an essential role in the development and metastasis of various tumors by mediating inflammatory responses (23, 30). Therefore, exploring the relevant molecular mechanisms of the STING pathway in PCa bone metastasis will bring new opportunities for the treatment of PCa bone metastasis.

The random forest algorithm is an excellent machine learning algorithm that has been commonly used in the biosciences field. Three key genes were finally screened in this study based on the MDA and MDG values assigned to each gene by this algorithm. The MDA indicates the extent to which model accuracy decreases when a variable is excluded, and the MDG indicates the extent to which a variable contributes to the reduction of the Gini in a random forest, with a higher Gini indicating a higher prediction error rate for that node (57). Therefore, the higher the two metrics are, the more critical their corresponding genes are. The subsequent comprehensive analysis demonstrated that the three key genes play critical roles in PCa development and metastasis.

TREX1 (Three Prime Repair Exonuclease 1) is a major cytoplasmic nuclease, prevalently expressed in mammalian cells, that acts primarily on double-stranded DNA, specializing in the excision of oligonucleotides that are mismatched at their 3′-ends. The enzyme is mainly involved in immune regulation and DNA damage repair in the body (58). Numerous studies have shown that loss-of-function mutations in TREX1 lead to abnormal accumulation of cytoplasmic DNA, which in turn over-activates the natural immune response and ultimately leads to the development of autoimmune diseases, including systemic lupus erythematosus and vascular diseases, retinopathy, and cerebral leukoencephalopathy (59, 60). Recent studies have shown that TREX1 also plays a key role in tumors (61). In breast and colon cancers, high expression of TREX1 leads to blockage of type I IFN pathway activation, inhibiting the anti-tumor immune response and the anti-tumor response of immune checkpoint inhibitors (61, 62). However, this phenomenon seems to be reversed in PCa, where it was shown that the expression level of TREX1 was not associated with the degree of anti-tumor immune response to Radiotherapy-induced activation of the type I IFN pathway in three PCa cell lines (63). In this study, TREX1 was found to be negatively correlated with the degree of infiltration of multiple PCa bone metastasis-promoting immune cells (including Regulatory T cell, Myeloid derived suppressor cell, and Plasmacytoid dendritic cell), and was negatively correlated with PD-1 and CD-200. In addition, high expression of TREX1 can prevent the activation of the Ecm-Receptor Interaction pathway, which plays a crucial role in prostate bone metastasis (38–40). Therefore, TREX1 is not only valuable in the prediction of PCa bone metastasis but also has great potential in its immunotherapy. The related molecular mechanisms deserve to be explored in depth.

RELA (RELA Proto-Oncogene, NF-KB Subunit) is a ubiquitous transcription factor involved in a variety of biological processes. Previous studies have shown that RELA plays a key role in the initial stages of Pca (64), and this study found that RELA has an important role in the carcinogenesis, development and metastasis of PCa. Correlation analysis showed that RELA was strongly associated with multiple prostate cancer driver genes (SPOP, AR, FOXA1, ATM, and PTEN); the Depmap database results showed that RELA had a high effect on the proliferation of PCa cell lines; bio-functional analyses showed that RELA may hinder the occurrence of bone metastasis by inhibiting the Ecm-Receptor Interaction pathway; subsequent cell and tissue experiments have also demonstrated that overexpression of RELA can significantly inhibited the proliferation and migration of PCA cells (DU145 and PC3), as well as expression of RELA is significantly decreased in bone metastasis; Immune analysis results showed that RELA was significantly negatively correlated with 2 types of bone metastasis-promoting immune cells (regulatory T cells and plasmacytoid dendritic cells), and significantly negatively correlated with PD-1 and CD-200. These results strongly suggest that RELA plays a crucial role in prostate cancer occurrence, progression and metastasis based on multiple molecular mechanisms. Therefore, RELA is likely to be a new therapeutic target for advanced PCa, and its complex biomolecular function is worthy of subsequent in-depth study.

As for CASP8 (Caspase 8), this gene is a member of the cysteine-aspartate protease (cysteinyl aspartate) family and plays a central role in apoptosis and necroptosis (65). Studies have shown that this gene is closely associated with PCa and its recurrence and can be used as a biomarker for bone metastasis in high-risk PCa (66, 67). This study found that CASP8 can activate three pathways, including Ecm-Receptor Interaction, Cell Cycle, and Homologous Recombination. In addition, CASP8 was positively associated with three types of pro-bone metastatic cells (Myeloid derived suppressor cell, Plasmacytoid dendritic cell, and Regulatory T cell) and two immune checkpoints (CD-200 and CTLA-4). Therefore, this gene is important in PCa risk prediction and bone metastasis prediction and also deserves attention.

At the end of this study, we constructed a nomogram and verified its reliability and accuracy in several approaches. This tool provides a reliable diagnostic tool for clinicians’ diagnosis and treatment. In addition, we also identified 22 potential targeted drugs of key genes with satisfactory molecular docking effects. In conclusion, this study mined three bone metastasis related key genes for PCa based on machine learning, explored their related molecular mechanisms, constructed a reliable nomogram and discovered 22 kinds of potential targeted drugs. This study can improve the treatment of advanced PCa patients and provides a theoretical basis for subsequent research.

Of course, this study has some limitations. First, this study is essentially a retrospective analysis, which needs to be corroborated by subsequent prospective studies; second, only one dataset was included in this study to construct the nomogram, and the generalization ability of the model performance needs to be validated by the subsequent including of more datasets; third, this study did not perform more in-depth basic experimental validation, which needs to be supplemented in subsequent studies; and finally, it is difficult to define a fixed threshold for the expression of various genes in the clinic, which poses an obstacle to the popularization of diagnostic tools.

## 5 Conclusion

In this study, we explored the relevant mechanisms of the STING pathway in PCa bone metastasis based on bioinformatics analysis techniques. Based on the random forest algorithm mined three key genes, and the critical roles of the key genes in PCa development, metastasis, and tumor immunity were explored by multiple analyses. Finally, based on the key genes, a reliable nomogram was constructed and potential targeted drugs were discovered. In conclusion, this study provides new therapeutic targets and a reliable diagnostic tool for clinical treatment and provides a theoretical basis for follow-up studies.

## Data availability statement

The original contributions presented in the study are included in the article/Supplementary material, further inquiries can be directed to the corresponding authors.

## Ethics statement

This study was approved by the Clinical Research Ethics Committee of the First Affiliated Hospital of Chongqing Medical University (ID: K2024-185-01). The studies were conducted in accordance with the local legislation and institutional requirements. The ethics committee/institutional review board waived the requirement of written informed consent for participation from the participants or the participants’ legal guardians/next of kin because the experiment was performed using pathological tissues left over from postoperative pathological examinations.

## Author contributions

GL: Conceptualization, Data curation, Formal analysis, Investigation, Methodology, Resources, Software, Validation, Visualization, Writing – original draft. RZ: Conceptualization, Data curation, Formal analysis, Investigation, Methodology, Resources, Software, Validation, Visualization, Writing – original draft. ZX: Conceptualization, Data curation, Formal analysis, Investigation, Resources, Validation, Visualization, Writing – original draft. XQ: Data curation, Investigation, Resources, Visualization, Writing – original draft. YD: Data curation, Investigation, Resources, Visualization, Writing – original draft. YZ: Data curation, Investigation, Resources, Visualization, Writing – original draft. HL: Data curation, Investigation, Resources, Visualization, Writing – original draft. DT: Methodology, Project administration, Supervision, Writing – review & editing. ZL: Funding acquisition, Methodology, Project administration, Supervision, Writing – review & editing. WH: Methodology, Project administration, Resources, Supervision, Writing – review & editing.

## Footnotes

^1^ www.ncbi.nlm.nih.gov/geo/

^2^ www.genecards.org

^3^ www.intogen.org/search

^4^ www.depmap.org/portal/

^5^ https://guolab.wchscu.cn/GSCA/#/

^6^ https://maayanlab.cloud/Enrichr/#

^7^ https://www.rcsb.org/

^8^ https://pubchem.ncbi.nlm.nih.gov/

## Supplementary material

SUPPLEMENTARY FIGURE 1 Removal of outliers based on clustering algorithm. **(A)** Sample clustering to detect outliers; **(B)** Sample dendrogram and trait heatmap.

SUPPLEMENTARY FIGURE 2 200 times 5-fold cross validation of 3 key genes and the nomogram.

SUPPLEMENTARY DATA SHEET 1 22 potential TREX1 and RELA targeted overexpression drugs.
