# Supplementary figures and images for: Mining bone metastasis related key genes of prostate cancer from the STING pathway based on machine learning
Source: Front Med (Lausanne). 2024 May 21;11:1372495. doi: 10.3389/fmed.2024.1372495 (PMC11148254; doi:10.3389/fmed.2024.1372495)

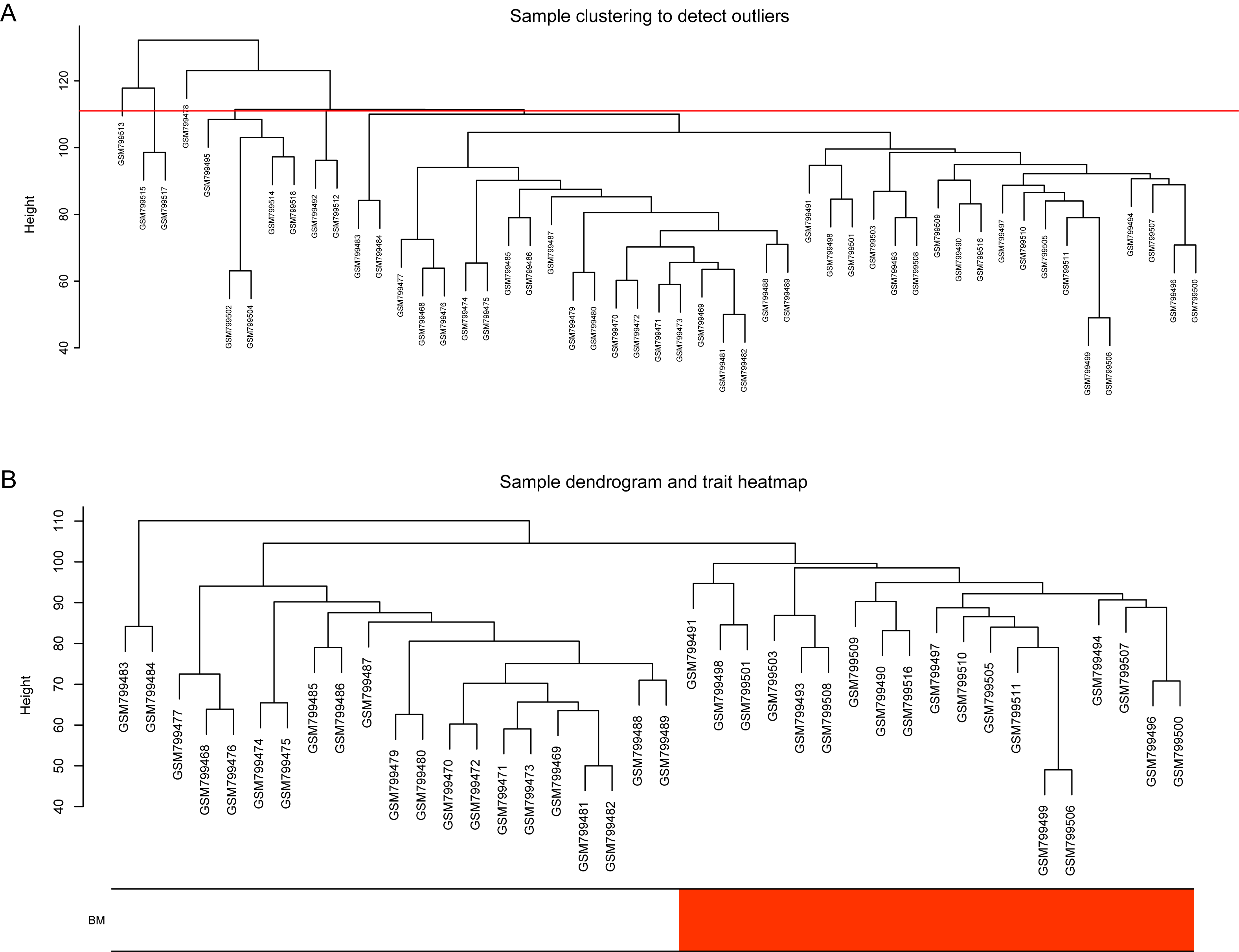

Supplement: SUPPLEMENTARY FIGURE 1 — Removal of outliers based on clustering algorithm. (A) Sample clustering to detect outliers; (B) Sample dendrogram and trait heatmap. [file Image_1.TIF]

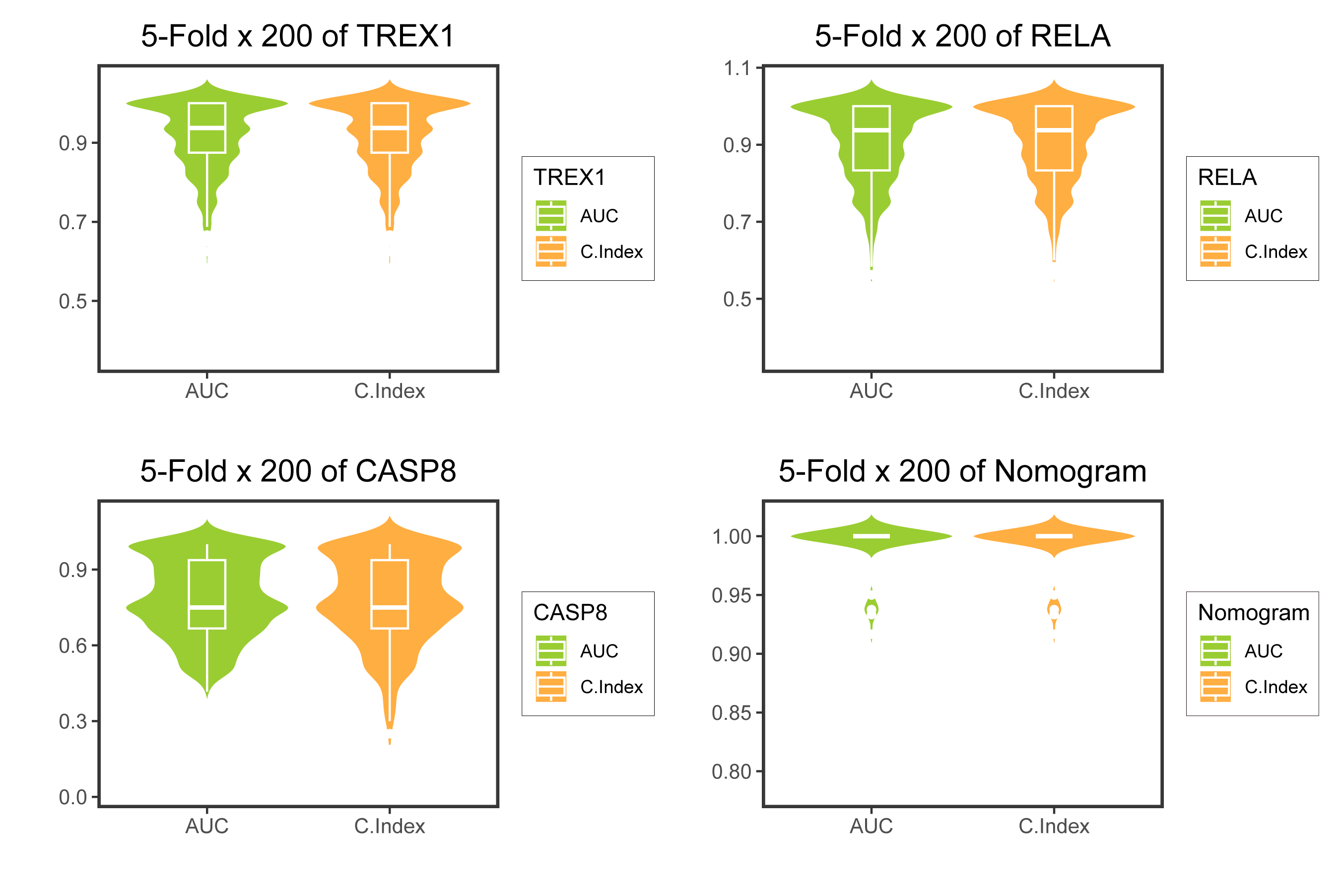

Supplement: SUPPLEMENTARY FIGURE 2 — 200 times 5-fold cross validation of 3 key genes and the nomogram. [file Image_2.TIF]
